# Patients and dermatologists are largely satisfied with ChatGPT-generated after-visit summaries: A pilot study

**DOI:** 10.1016/j.jdin.2023.12.004

**Published:** 2023-12-29

**Authors:** Albert T. Young, Brittany N. Lane, David Ozog, Natalie H. Matthews

**Affiliations:** a Department of Dermatology, Henry Ford Hospital, Detroit, Michigan; b Department of Medicine, Michigan State University College of Human Medicine, East Lansing, Michigan

**Keywords:** after-visit summary, artificial intelligence, ChatGPT, large language model, machine learning, patient education

*To the Editor:* ChatGPT, an artificial intelligence–driven large language model (LLM) that generates human-like text in a conversational manner, is increasingly being used in medicine.^1^^,^^2^ Its potential to enhance patient after-visit summary (AVS) has not been explored.

Dermatologists from a single academic institution wrote AVS using a standardized prompt for ChatGPT. The physicians input diagnosis and treatment. No identifying information was provided. The prompt included etiopathogenesis, symptoms, when to seek treatment, management, procedure risks, medication side effects, and prioritized resources from credible medical organizations (example prompt Fig 1). For comprehension, instructions were output at a sixth-grade reading level. Inaccuracies were corrected by the physician before the patient received the AVS (example AVS output Supplementary Fig 1, available via Mendeley at https://data.mendeley.com/datasets/ppfgpd82s8/1) with a survey. AVS was generated for 53 patients (72% females; age range, 1-81 years), representing 60 rare diagnoses and 8 in-office procedures. GPT-3.5 was used because it is freely available and allows unlimited queries.^3^

**Fig 1:**
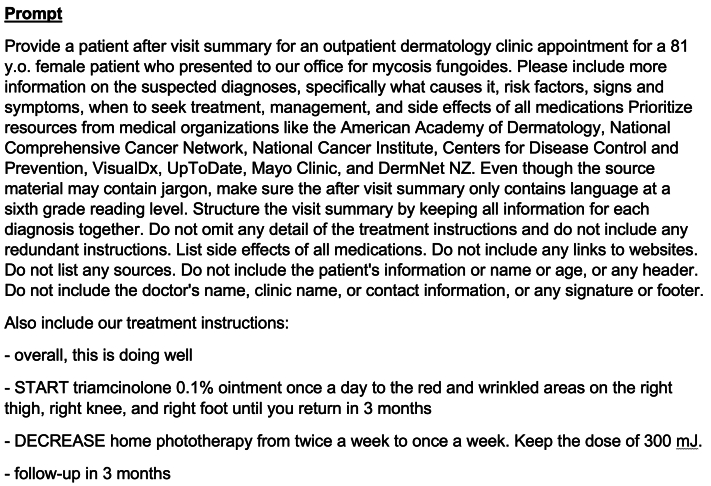
Sample prompt to ChatGPT.

On a 5-point Likert-type scale, patients reported high satisfaction with the AVS overall (mean, 4.91), AVS was easy to understand (mean, 4.89), and AVS summarized the appointment well (mean, 4.92); 96% of patients would recommend the AVS to others (Table I). Compared with prior AVS, 18 (34%) patients found it somewhat to very different.

**Table I:** Patient survey responses regarding ChatGPT-enhanced after-visit summaries

Question	1	2	3	4	5	Missing
How easy was it to understand the information provided in your after-visit summary?	Not at all satisfied	Somewhat dissatisfied	Neutral	Somewhat satisfied	Very satisfied	
	*n* *=* 0 (0.0%)	*n* = 0 (0.0%)	*n* = 0 (0.0%)	*n* = 5 (9.4%)	*n* = 48 (90.6%)	*n* = 0 (0.0%)
How easy was it to understand the information provided in your after-visit summary?	Very difficult	Somewhat difficult	Neutral	Somewhat easy	Very easy	
	*n* = 0 (0.0%)	*n* = 0 (0.0%)	*n* = 1 (1.9%)	*n* = 4 (7.5%)	*n* = 48 (90.6%)	*n* = 0 (0.0%)
How well did the after-visit summary summarize the information provided during your appointment?	Very poorly	Somewhat poorly	Neutral	Somewhat well	Very well	
	*n* = 0 (0.0%)	*n* = 0 (0.0%)	*n* *=* 0 (0.0%)	*n* *=* 4 (7.5%)	*n* *=* 49 (92.4%)	*n* *=* 0 (0.0%)
How does this after-visit summary compare with prior after-visit summaries that you have received?	Very similar	Somewhat similar	Neutral	Somewhat different	Very different	
	*n* *=* 13 (24.5%)	*n* *=* 5 (9.4%)	*n* *=* 16 (30.2%)	*n* *=* 10 (18.9%)	*n* *=* 8 (15.1%)	*n* *=* 1 (1.9%)
Would you recommend this after-visit summary to others?				No	Yes	
				*n* *=* 2 (3.8%)	*n* *=* 50 (94.3%)	*n* *=* 1 (1.9%)

Based on 10 free text responses, patients reported that their ChatGPT-enhanced AVS was more detailed (*n* *=* 5), better structured (*n* *=* 4), easier to understand (*n* *=* 3), and more concise (*n* *=* 1) than previous AVS. One patient found that ChatGPT-enhanced AVS was less detailed (*n* *=* 1), and another found it “a little grammatically awkward, but clear enough” (*n* *=* 1). Based on 27 free text responses, most patients did not feel that any changes were needed to the AVS (*n* *=* 26); one asked for main points to be bolded (*n* *=* 1).

Ten of 53 (19%) AVS required editing by physicians owing to incorrect or incomplete information. Incorrect recommendations by ChatGPT included routine use of over-the-counter topical antibiotics for wound care, loss of specificity of anatomic sites to be treated (eg, directions to trim the left great toenail was changed to “toenails”), and unpredictable omission of medication side effects (eg, risk of ochronosis with hydroquinone).

Artificial intelligence capabilities are rapidly growing—Med-PaLM 2, another LLM, generated answers to consumer medical questions that blinded physician raters preferred over physician-generated answers.^4^ ChatGPT has also been studied in melanoma care.^5^

During prompt engineering, it was helpful to include precise instructions regarding desired format and content and to avoid listing sources (to discourage ChatGPT from “hallucinating” inaccurate sources). Jargon not desired in the output should be excluded from the prompt because ChatGPT tended to repeat phrases from the prompt in the output.

Limitations of this study include small sample size, single center institution, and use of an unvalidated scale to assess satisfaction. Future study is needed to evaluate other LLMs including GPT-4.0.

Overall, patients viewed ChatGPT-enhanced AVS favorably. Important omissions and inaccuracies were corrected by physicians, emphasizing the need for provider oversight and continued optimization of prompt selection. This study highlights the necessity of recognizing the potential benefits and harms of LLMs for health care professionals.

## Conflicts of interest

None disclosed.
